# Daily rhythmicity of clock gene transcript levels in fast and slow muscle fibers from Chinese perch (*Siniperca chuatsi*)

**DOI:** 10.1186/s12864-016-3373-z

**Published:** 2016-12-08

**Authors:** Ping Wu, Yu-Long Li, Jia Cheng, Lin Chen, Xin Zhu, Zhi-Guo Feng, Jian-She Zhang, Wu-Ying Chu

**Affiliations:** 1 Department of Bioengineering and Environmental Science, Changsha University, Changsha, Hunan 410003 China; 2 Collaborative Innovation Center for Efficient and Health Production of Fisheries in Hunan Province, Changde, 415000 China; 3 College of Life Sciences, Xinyang Normal University, Xinyang, Henan 464000 China

**Keywords:** Chinese perch, Clock genes, Skeletal muscle, Daily rhythmicity, Myogenic regulatory factors

## Abstract

**Background:**

Clock genes are considered to be the molecular core of biological clock in vertebrates and they are directly involved in the regulation of daily rhythms in vertebrate tissues such as skeletal muscles. Fish myotomes are composed of anatomically segregated fast and slow muscle fibers that possess different metabolic and contractile properties. To date, there is no report on the characterization of the circadian clock system components of slow muscles in fish.

**Results:**

In the present study, the molecular clock components (*clock*, *arntl1/2*, *cry1*/*2*/*3*, *cry-dash*, *npas2*, *nr1d1*/*2*, *per1*/*2*/*3*, *rorα* and *tim* genes) and their daily transcription levels were characterized in slow and fast muscles of Chinese perch (*Siniperca chuatsi*). Among the 15 clock genes, *nrld2* and *per3* had no daily rhythmicity in slow muscles, and *cry2*/*3* and *tim* displayed no daily rhythmicity in fast muscles of the adult fish. In the slow muscles, the highest expression of the most clock paralogs occurred at the dark period except *arntl1*, *nr1d1*, *nr1d2* and *tim*. With the exception of *nr1d2* and *tim*, the other clock genes had an acrophase at the light period in fast muscles. The circadian expression of the myogenic regulatory factors (*mrf4* and *myf5*), *mstn* and *pnca* showed either a positive or a negative correlation with the transcription pattern of the clock genes in both types of muscles.

**Conclusions:**

It was the first report to unravel the molecular clock components of the slow and fast muscles in vertebrates. The expressional pattern differences of the clock genes between the two types of muscle fibers suggest that the clock system may play key roles on muscle type-specific tissue maintenance and function.

**Electronic supplementary material:**

The online version of this article (doi:10.1186/s12864-016-3373-z) contains supplementary material, which is available to authorized users.

## Background

Fish skeletal muscles are the most abundant tissue in the body mass and play an important role in the process of certain physiological metabolism [1]. Similar to other peripheral tissues of the body, skeletal muscles have circadian rhythms [2]. These rhythms are regulated by a transcriptional-translational and post-translational feedback network termed as the molecular clock [3]. There are several major components in the molecular clock, including circadian locomotor output cycles kaput (*clock*)*,* aryl hydrocarbon receptor nuclear translocator-like protein 1 (*arntl1* or *bmal1*)*,* cryptochrome (*cry*), and period protein (*per*) etc. [4]. Two transcriptional activation proteins of the molecular clock, namely *clock* and *arntl1,* are the basic-helix-loop-helix (bHLH) transcription factors that form into a heterodimer in the nucleus. Together, they transactivate *per* and *cry* gene expression via binding to the E-box elements (CACGTG) at their promoter sequences [5–7]. *Per* and *cry* then translocate into the cell nucleus, in which they inactivate *clock* and *arntl* activity, thereby repressing their own transcription. The clock mechanism plays a pivotal role in myogenesis, gene transcription, and maintenance of muscle metabolism [8, 9]. The molecular clock components have been identified in skeletal muscles and showed a circadian rhythms of expression. In addition to these clock genes, more than 2300 other genes have circadian pattern of expression in skeletal muscles. These muscle genes with circadian pattern of expression are believed to be regulated by the major molecular clock genes and the clock-controlled transcription factors [10].

Fish are excellent model species for investigating the regulation of skeletal muscle physiology in vertebrates because it has several structural features making convenient to experimental analysis [11, 12]. The slow-contracting red and fast-contracting white muscles are the two main muscle fiber types in fish [13, 14]. Particularly, they are localized into physically distinct area of the fish body. The fast muscle fibers are the main component of skeletal muscles that distribute along the spine of the whole body. Their explosive force is strong for fast swimming using energy from glycolysis [15, 16]. Slow fibers, on the other hand, contain high contents of mitochondria and their metabolism is completely aerobic [16, 17]. These unique features enable slow fibers to maintain sustained swimming and support oxygen respiration [18].

Recently, several studies have been reported on the clock rhythmicity in fish. In zebrafish, the major clock genes showed similar circadian expression patterns in fast muscles compared with the central organs, such as retina and brain [19, 20]. It has been shown that insulin-like growth factor binding proteins (*igfbp3* and *igfbp5b*) and myogenic regulatory factor 4(*mrf4*) were controlled by the core clock genes in zebrafish skeletal muscles [20, 21]. In Atlantic cod fast muscles, similar circadian clock system has been identified [2]. Another myogenic regulatory factor *myf5* exhibited a significant correlation with the core clock genes at the transcription levels [2]. However there is little information on the circadian clock system components in fish slow muscle. The Chinese perch (*Siniperca chuatsi*) is one of the most important species in aquaculture in China [22]. Its high nutritional value, high protein content and appealing taste have led to its expanded large-scale aquaculture in china [23]. In the present study, we report the characterization of circadian clock system in both fast and slow muscles of Chinese perch, and the correlation analysis between core clock gene expression and 11 myogenic related genes in the two types of muscles.

## Results

### Isolation of Chinese perch clock genes and their molecular characteristics

**Fig. 1:**
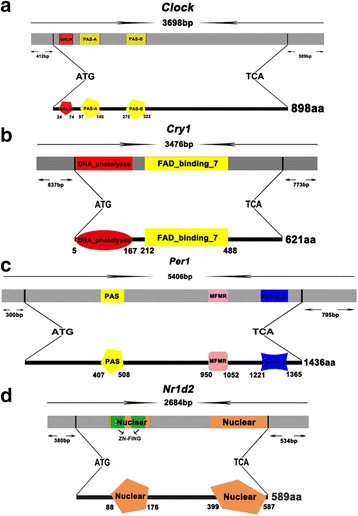
Schematic representation of the Chinese perch clock gene structure: (**a**) *Clock*; (**b**) *Cry*; (**c**) *Per1*; (**d**) *Nr1d2*. The predicted domain structure of *clock*, *cry1*, *per1* and *nr1d2* are shown as above and presented below the sequence structure. The split domain of clock proteins is shown in different colors. Both *clock* and *per1* proteins have a PAS domain. cry1 includes a photosensitive domain of DNA photolyase, and *nr1d2* has a unique zinc finger structure that connects with the nuclear DNA

A total of 4 complete and 11 partial sequences of 15 clock genes were cloned from the skeletal muscles of Chinese perch. These include *arntl1*, *arntl2*, *clock*, *cry1*, *cry2*, *cry3*, *cry-dash*, *npas2*, *nr1d1*, *nr1d2*, *per1*, *per2*, *per3*, *rorα* and *tim* (Additional file 1). The full-length cDNAs of *clock*, *cry1*, *per1* and *nr1d2* were 3698 bp, 3476 bp, 5406 bp and 2684 bp, respectively (Fig. 1). The *clock* gene contained the 5′-non-coding region (5′-UTR) of 412 bp, an open reading frame (ORF) of 2697 bp and the 3’-non-coding region (3′-UTR) of 589 bp. The *cry1* gene contained the 5′-UTR of 837 bp, an ORF of 1866 bp and the 3′-UTR of 773 bp. The *per1* gene included the 5′-UTR of 300 bp, an ORF of 4311 bp and the 3′-UTR of 795 bp. The *nr1d2* gene contained the 5′-UTR of 380 bp, an ORF of 1770 bp and the 3′-UTR of 534 bp.

**Fig. 2:**
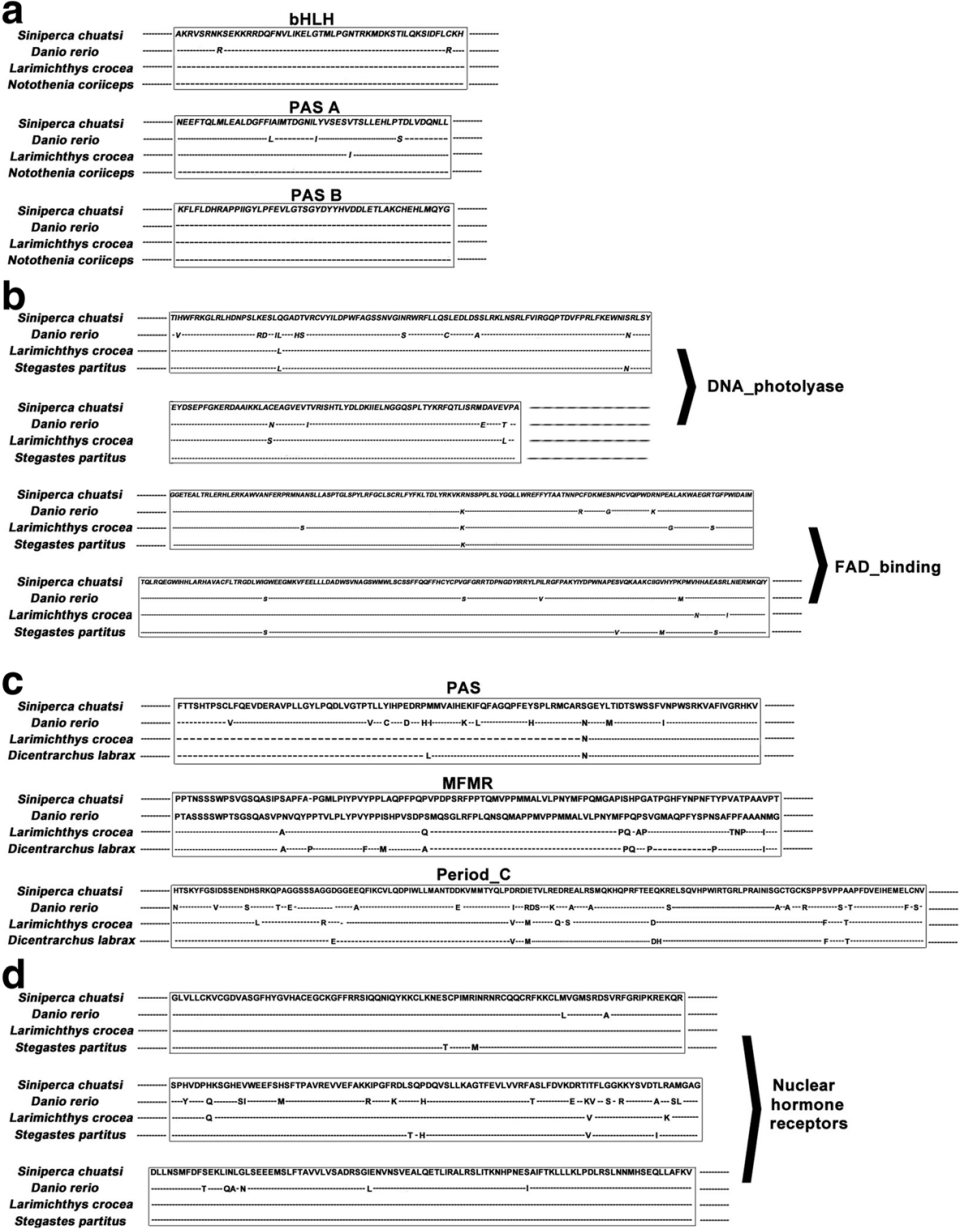
Amino acid Sequences alignment of functional domains of Chinese Perch 4 clock proteins with the homologous clock proteins of other species: (**a**) *Clock*; (**b**) *Cry1*; (**c**) *Per1*; (**d**) *Nr1d2*. The box indicates the functional regions of clock proteins

The conserved structural and functional domains were characterized based on the predicted protein sequences. The *clock* protein contains the conserved bHLH, Per-Arnt-Ser (PAS) A and PAS B domains. The amino acid sequences of these domains showed 100%, 98% and 100% similarities to those of *Larimichthys crocea*, respectively (Figs. 1 and 2). The *per1* has a PAS domain, a G-box binding protein multifunctional mosaic region (MFMR) and a 2/3 C-terminal region of *period* protein. The amino acid sequences of these domains showed 99%, 91% and 93% similarities to those of *L. crocea*, respectively. The *cry1* contains two functional domains, a flavin adenine dinucleotide (FAD)-binding domain and a DNA photolyase domain. The amino acid sequences of these two domains showed 98% and 98% similarities to those of *L. crocea,* respectively. The *nr1d2* has two nuclear hormone receptors DNA-binding domains and the amino acid sequences of these domains showed 100% and 98% similarities to those of *L. crocea*, respectively.

### The rhythmicity of clock genes during a daily cycle in fast and slow muscles

**Fig. 3:**
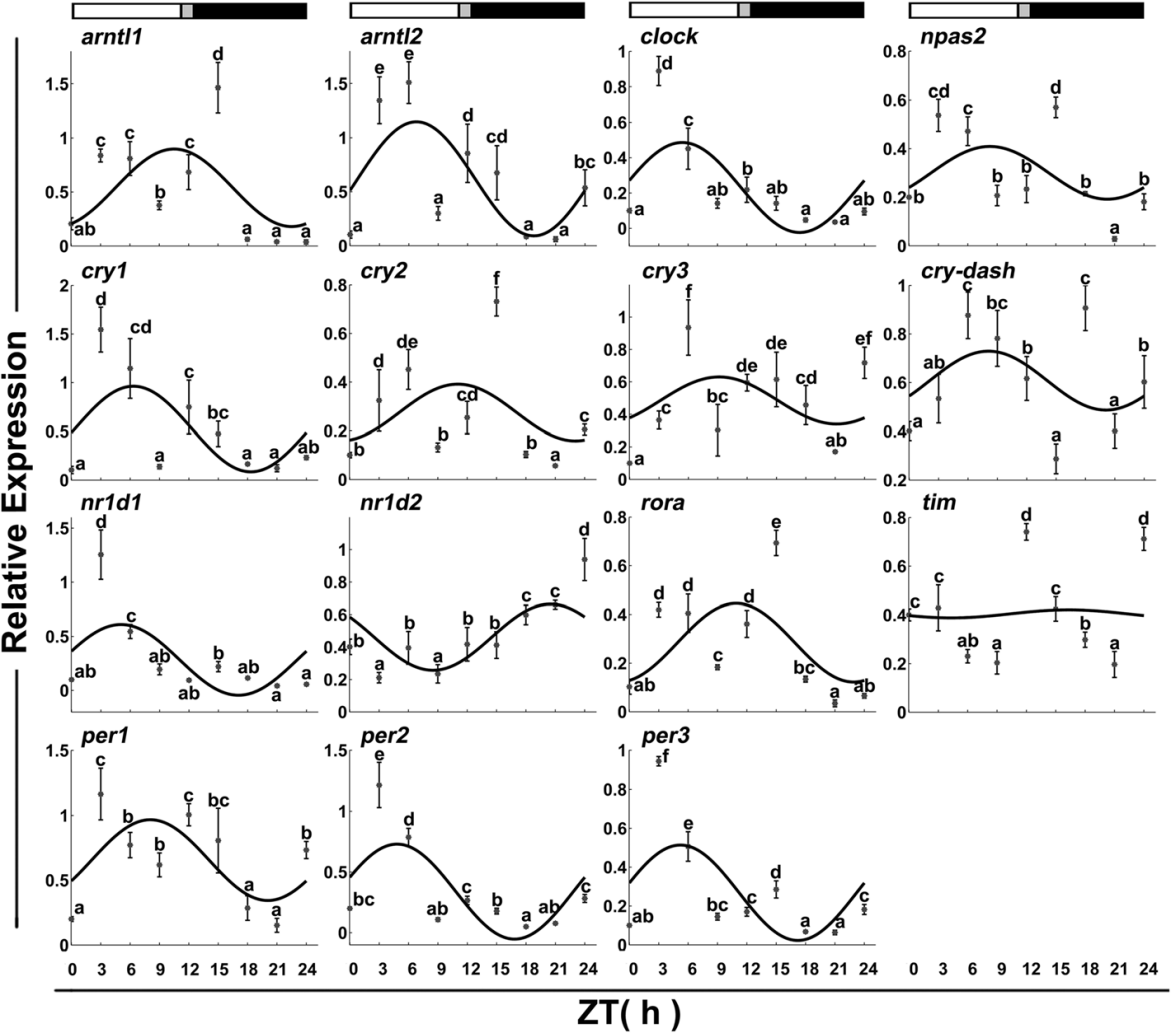
Expression of clock genes in fast skeletal muscles during a daily cycle. The values are mean ± SEM (*n* = 6) of the normalized transcript levels of each clock gene. Significant differences between time points are indicated by different lower-case letters. The line represents the periodic sinusoidal function of gene expression in a circadian cycle constructed from the periodicity parameters calculated using COSINOR. The photoperiod regime is represented by the composite block. *White*, *black* and *gray*, represent the *light*, *dark* and *light–dark* transition phases, respectively

**Fig. 4:**
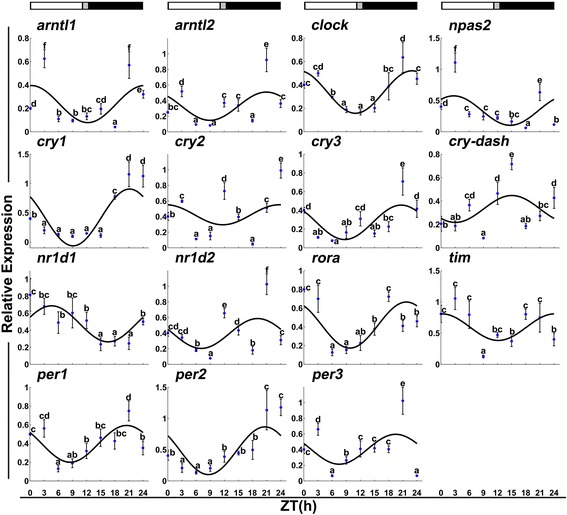
Expression of clock genes in slow skeletal muscles during a daily cycle. The values are mean ± SEM (*n* = 6) of the normalized transcript levels of each clock gene. Significant differences between time points are indicated by different lower-case letters. The line represents the periodic sinusoidal function of gene expression in a circadian cycle constructed from the periodicity parameters calculated using COSINOR. The photoperiod regime is represented by the composite block. *White*, *black* and *gray*, represent the *light*, *dark* and *light–dark* transition phases, respectively

The expression pattern of the clock genes was determined for daily rhythmicity in fast and slow muscle (Tables 1 and 2, and Figs. 3 and 4). Among the 15 clock genes, *nr1d2* and *per3* have no daily rhythmicity in slow muscles, while *cry3*, *npas2*, and *tim* have no rhythmicity in fast muscle. *Cry2* and *cry-dash* have no daily rhythmicity in neither slow nor fast muscles. In contrast, *arntl1*, *arntl2*, *clock*, *cry1*, *per1*, *per2*, *nr1d1* and *rorα* displayed the daily rhythmicity in both fast and slow skeletal muscles. In fast muscles, transcriptional activation factors *arntl1*, *arntl2* and *clock* displayed the daily rhythmicity with an acrophase during the light phase (Fig. 3). There was no much temporal difference between *arntl2* and *clock* expression. In slow skeletal muscles, *arntl1*, *arntl2* and *clock* still displayed the daily rhythmicity but *arntl1* had an acrophase during the dark phase (Fig. 4). The temporal expression of *arntl1* and *clock* showed no apparent difference. Finally, *npas2* showed the daily rhythmicity with an acrophase during the light phase only in slow muscles (Fig. 4).

**Table 1:** Rhythmicity parameters of clock genes and muscle-related genes transcription in Chinese perch fast skeletal muscle

Gene name	Amplitude	*P* value	Mesor	Acrophase	ZT(h)
arntl1	0.36	**0.21**	0.54	2.75	10.52
arntl2	0.53	**0.07**	0.62	1.77	6.77
cry1	0.44	**0.17**	0.52	1.66	6.33
cry2	0.12	0.46	0.27	2.89	11.05
cry3	0.15	0.49	0.48	2.39	9.14
npas2	0.11	0.45	0.30	2.16	8.26
nr1d1	0.33	**0.17**	0.28	1.32	5.05
nr1d2	0.20	**0.10**	0.46	5.37	20.51
per1	0.31	**0.13**	0.66	2.11	8.07
per2	0.39	**0.06**	0.34	1.26	4.83
per3	0.25	**0.16**	0.27	1.36	5.21
rorα	0.16	**0.20**	0.28	2.86	10.91
tim	0.02	0.98	0.40	4.27	16.33
clock	0.25	**0.10**	0.23	1.41	5.40
crydash	0.12	0.49	0.61	2.13	8.09
foxk2	0.07	0.89	0.31	3.82	14.57
mbnl1	1.30	**0.07**	1.02	1.63	6.22
mrf4	0.26	**0.04**	0.55	2.82	10.77
mstn	0.22	**0.21**	0.27	0.88	3.35
murf1	0.04	0.91	0.30	3.52	13.46
myf5	0.20	**0.26**	0.30	0.70	2.66
myoD	0.37	**0.04**	0.51	1.21	4.63
myoG	0.30	**0.01**	0.29	1.47	5.61
pdk4	0.17	0.48	0.39	1.09	4.18
pcna	0.29	**0.05**	0.32	6.00	22.91
ucp3	0.05	0.91	0.28	3.38	12.92

*Note*: Expression levels of clock genes and muscle-related genes are highlighted in bold while they displayed daily rhythmicity. The *P* value is defined as the noise/signal ratio of the oscillation amplitude. Daily rhythmicity is indicated when *P* value is less than 0.3

**Table 2:** Rhythmicity parameters of clock genes and muscle-related genes transcription in Chinese perch slow skeletal muscle

Gene name	Amplitude	*P* value	Mesor	Acrophase	ZT(h)
arntl1	0.16	**0.18**	0.24	0.06	0.22
arntl2	0.18	**0.29**	0.33	5.49	20.96
cry1	0.49	**0.01**	0.42	5.53	21.12
cry2	0.13	0.64	0.42	6.19	23.65
cry3	0.18	**0.084**	0.27	5.41	20.66
npas2	0.24	**0.25**	0.34	0.71	2.69
nr1d1	0.21	**0.04**	0.48	1.19	4.56
nr1d2	0.19	0.37	0.40	4.99	19.08
per1	0.20	**0.03**	0.40	5.37	20.53
per2	0.38	**0.05**	0.48	5.39	20.60
per3	0.19	0.38	0.40	5.10	19.50
rorα	0.25	**0.04**	0.42	5.69	21.75
tim	0.21	**0.21**	0.60	0.04	0.16
clock	0.18	**0.01**	0.34	6.01	22.94
crydash	0.12	0.41	0.33	3.93	15.03
foxk2	0.14	**0.29**	0.23	5.58	21.32
mbnl1	0.19	0.31	0.31	5.44	20.77
mrf4	0.09	**0.29**	0.40	0.70	2.67
mstn	0.23	**0.28**	0.34	0.87	3.32
murf1	0.27	0.31	0.35	5.33	20.34
myf5	0.19	**0.09**	0.31	5.56	21.23
myoD	0.13	0.42	0.45	5.73	21.90
myoG	0.16	0.32	0.45	0.74	2.81
pdk4	0.16	0.34	0.31	5.96	22.78
pcna	0.26	**0.02**	0.35	5.25	20.05
ucp3	0.05	0.95	0.39	1.16	4.45

*Note*: Expression levels of clock genes and muscle-related genes are highlighted in bold while they displayed daily rhythmicity. The *P* value is defined as the noise/signal ratio of the oscillation amplitude. Daily rhythmicity is indicated when *P* value is less than 0.3

The transcriptional repressors *cry2*, *cry3* and *tim* were arrhythmic but *cry1*, *per1*, *per2* and *per3* were rhythmic in fast muscles. With the exception of *tim*, the other clock genes were expressed with the acrophase during the light phase in fast muscles. In slow muscles, the *cry2* and *per3* were arrhythmic but *cry1*, *cry3*, *per1*, *per2* and *tim* were rhythmic. The *tim* was highly expressed during the light phase with the acrophase at Zeitgeber time (ZT) = 0.16 h, but the other genes were expressed with the acrophase during the dark phase in slow muscles.

The nuclear receptors *rorα*, *nr1d1* and *nr1d2* displayed the daily rhythmicity in fast muscles. *rorα* and *nr1d1* had an acrophase during the light phase (ZT = 5.05 h) but *nr1d2* had an acrophase during the dark phase (ZT = 20.51 h). In slow muscles, *rorα* and *nr1d1* exhibited a daily rhythmic expression but *nr1d2* was arrhythmic. The *rorα* and *nr1d1* genes had an acrophase during the light phase, but *nr1d2* exhibited a similar process during the dark phase (ZT = 19.08 h).

In fast muscles, the mRNA transcript levels of *clock* were positively correlated with the expression pattern of *per2* and *per3* in fast muscles with a higher correlation index (*r* > 0.8; Table 3). *Arntl2* also exhibited a positive correlation with *per2* and *per3*. N*r1d2*, however, showed a moderate negative correlation to *arntl1*, *clock*, *npas2*, *nr1d1*and *rorα*. In slow muscles,*clock* showed a moderate positive correlation to *per2* and *per3. Arntl2* also displayed a moderate positive correlation with *per2* expression. *Nr1d2* showed no correlation with other genes (Table 4).

**Table 3:** The expression correlations among different clock genes in fast muscle during a daily cycle

Correlations	Gene	Gene	*r*
Positive correlation	arntl1	arntl2	+0.64
	arntl1	cry1	+0.58
	arntl1	cry2	+**0.93**
	arntl1	npas2	+**0.88**
	arntl1	per1	+0.66
	arntl1	per3	+0.53
	arntl1	rorα	+**0.99**
	arntl2	cry1	+**0.94**
	arntl2	cry2	+0.61
	arntl2	cry3	+0.66
	arntl2	npas2	+0.76
	arntl2	nr1d1	+0.74
	arntl2	per1	+**0.83**
	arntl2	per2	+**0.86**
	arntl2	per3	+**0.83**
	arntl2	clock	+**0.82**
	arntl2	rorα	+0.64
	clock	npas2	+0.68
	clock	per1	+0.72
	clock	per2	+**0.98**
	clock	per3	+**0.98**
	clock	nr1d1	+**0.98**
	clock	cry1	+**0.94**
	cry1	npas2	+0.74
	cry1	nr1d1	+**0.89**
	cry1	per1	+0.79
	cry1	per2	+**0.94**
	cry1	per3	+**0.93**
	cry1	rorα	+0.60
	cry2	cry3	+0.60
	cry2	npas2	+**0.87**
	cry2	per1	+0.58
	cry2	rorα	+**0.94**
	cry3	per1	+0.54
	npas2	nr1d1	+0.69
	npas2	per1	+0.69
	npas2	per2	+0.66
	npas2	per3	+0.76
	npas2	rorα	+**0.90**
	nr1d1	per1	+0.63
	nr1d1	per2	+**0.95**
	nr1d1	per3	+**0.98**
	per1	tim	+0.50
	per1	per2	+0.68
	per1	per3	+0.72
	per1	rorα	+0.67
	per2	per3	+**0.97**
	per3	rorα	+0.53
Negative correlation	arntl1	nr1d2	−0.55
	clock	nr1d2	−0.55
	npas2	nr1d2	−0.52
	nr1d1	nr1d2	−0.56
	nr1d2	rorα	−0.52

*Note*: Only correlations with *r* > +0.5 or *r* < −0.5 and including at least one gene with significant daily rhythmicity are shown in this table. The following values were set to define the degree of correlation: data are moderately correlated if 0.5 < *r* < 0.79 and there is a strong correlation when *r* ≥ 0.80 which are highlighted in bold

**Table 4:** The expression correlations among different clock genes in slow muscle during a daily cycle

Correlations	Gene	Gene	*r*
Positive correlation	arntl1	arntl2	**0.84**
	arntl1	cry2	0.51
	arntl1	clock	0.77
	arntl1	npas2	**0.84**
	arntl1	nr1d2	0.55
	arntl1	per1	0.74
	arntl1	per3	0.68
	arntl2	clock	0.70
	arntl2	cry1	0.53
	arntl2	cry2	0.50
	arntl2	cry3	0.73
	arntl2	npas2	0.56
	arntl2	nr1d2	**0.89**
	arntl2	per1	**0.85**
	arntl2	per2	0.62
	arntl2	per3	**0.84**
	clock	cry1	0.75
	clock	cry3	0.62
	clock	npas2	0.56
	clock	per1	0.74
	clock	per2	0.61
	clock	per3	0.58
	clock	rorα	0.54
	clock	tim	0.62
	cry1	per1	0.51
	cry1	per2	**0.92**
	cry2	per2	0.58
	cry3	nr1d2	0.78
	cry3	per1	0.64
	cry3	per2	**0.83**
	cry3	per3	0.55
	npas2	per1	0.54
	npas2	per3	0.62
	npas2	tim	0.63
	nr1d2	per1	0.72
	nr1d2	per2	0.56
	per1	per2	0.51
	per1	per3	**0.88**
	per1	rorα	0.61
	rorα	tim	0.61

*Note*: Only correlations with *r* > +0.5 or *r* < −0.5 and including at least one gene with significant daily rhythmicity are shown in this table. The following values were set to define the degree of correlation: data are moderately correlated if 0.5 < *r* < 0.79 and there is a strong correlation when *r* ≥ 0.80 which are highlighted in bold

### Daily expression of myogenic related genes and the correlation with the clock components in fast and slow muscles

**Fig. 5:**
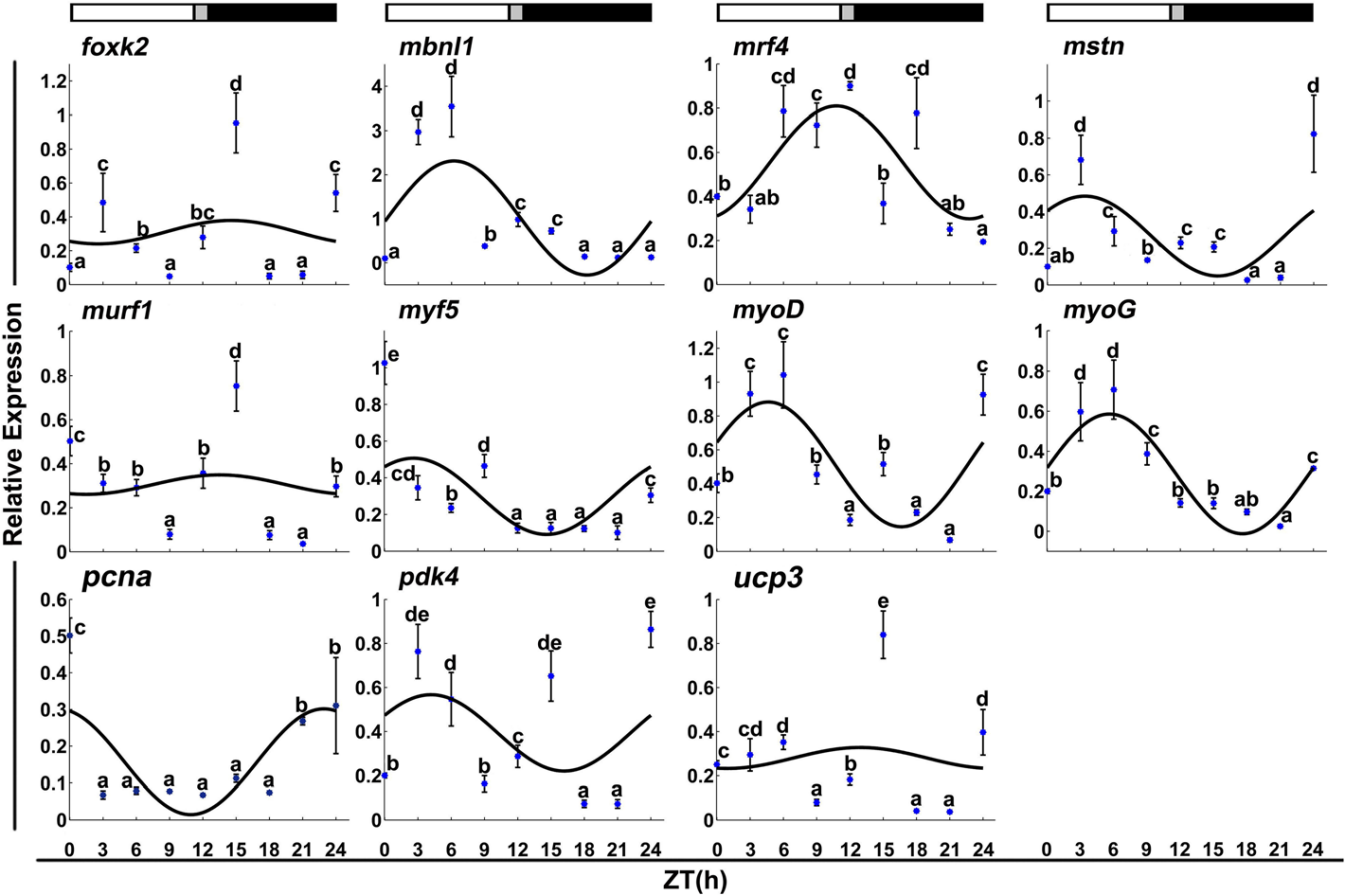
Expression of myogenesis-related genes in fast skeletal muscles during a daily cycle. The values are mean ± SEM (*n* = 6) of the normalized transcript levels of each clock gene. Significant differences between time points are indicated by different letter notations. The line represents the periodic sinusoidal function of gene expression in a circadian cycle constructed from the periodicity parameters calculated using COSINOR. The photoperiod regime is represented by the composite block above the graph. *White*, *black* and *gray* represent the *light*, the *dark* and the *light–dark* transition phases, respectively

**Fig. 6:**
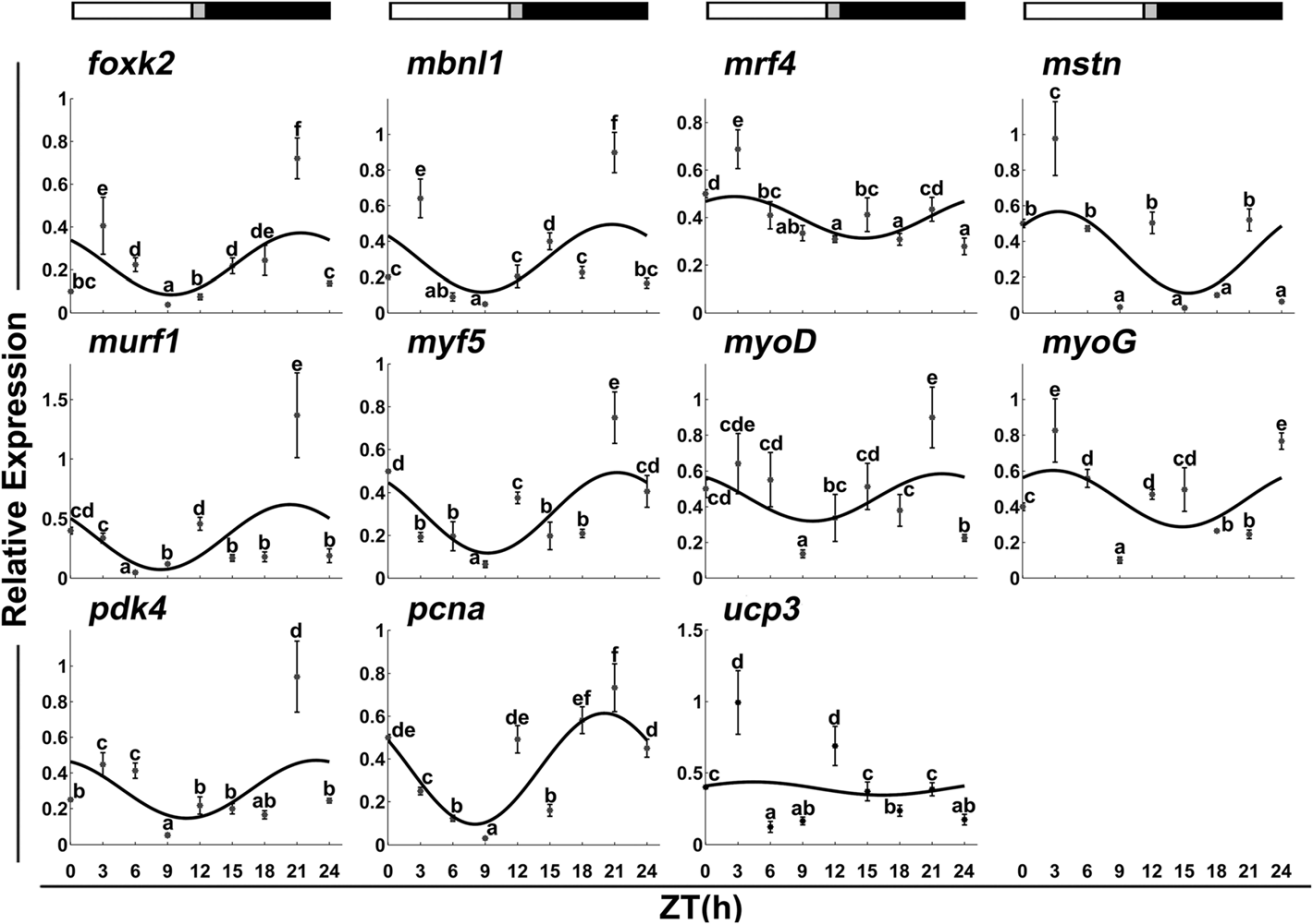
Expression of myogenesis-related genes in slow skeletal muscles during a daily cycle. The values are mean ± SEM (*n* = 6) of the normalized transcript levels of each clock gene. Significant differences between time points are indicated by different letter notations. The line represents the periodic sinusoidal function of gene expression in a circadian cycle constructed from the periodicity parameters calculated using COSINOR. The photoperiod regime is represented by the composite block above the graph. *White*, *black* and *gray* represent the *light*, the *dark* and the *light–dark* transition phases, respectively

In fast muscles, *mbnl1*, *mrf4*, *mstn*, *myf5*, *myoD*, *myoG* and *pcna* displayed a daily rhythmic expression (Table 1 and Fig. 5), but only *pcna* had an acrophase during the dark phase (ZT = 22.91 h). In slow muscles, *foxk2*, *mrf4*, *mstn*, *myf5* and *pcna* exhibited a daily rhythmic expression (Table 2 and Fig. 6). *Mrf4* and *mstn* showed an acrophase during the light phase (ZT = 2.67 h and 3.32 h). In contrast, *foxk2* (ZT = 21.32 h), *myf5* (ZT = 21.23 h) and *pcna* (ZT = 20.05 h) had an acrophase during the dark phase.

The mRNA transcript levels of most myogenic related genes had either a positive or a negative correlation with the daily expression of clock genes in fast and slow muscles (Tables 5 and 6). In fast muscles, the transcript levels of *pcna* displayed a moderate negative correlation with *arntl2*, *cry1* and *per1* (−0.8 < *r* < −0.5). *Mbnl1* showed a strong positive correlation with the daily expression of *arntl2*, *cry1*, *nr1d1*, *per2*, *per3* and *clock* (*r* > 0.8). *MyoG* was also positively correlated with *arntl2* and *per2*. In slow muscles, the transcript levels of *pcna* showed a moderate positive correlation with *arntl2*, *cry1* and *per1. myf5*, *mrf4*, *pcna* and *mstn* showed a strong positive correlation with *cry3* and *npas2* in slow muscles. The *foxk2* gene also showed a strong correlation with transcriptional activators, such as arm *arntl2*.

**Table 5:**
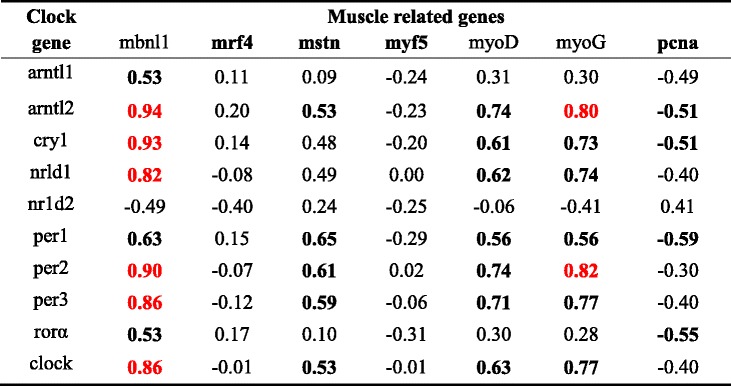
Correlation of expression levels of rhythmic clock and muscle-related genes in the fast muscle

*Note*: The following values were set to define the degree of correlation: data are considered to be moderately correlated if 0.5 < *r* < 0.79 or −0.79 < *r* < −0.5 and there is a strong correlation when *r* ≥ 0.80. And moderate correlation is marked in bold and strong correlation in red color

**Table 6:**
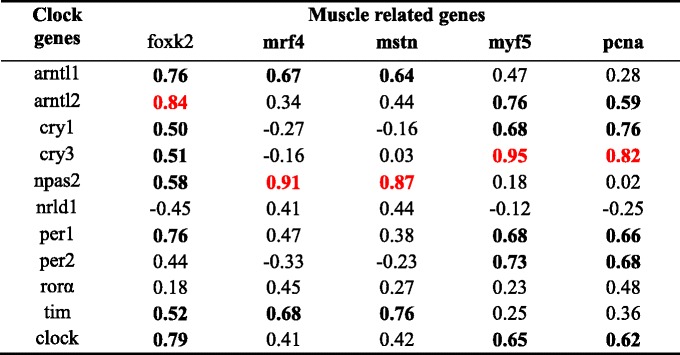
Correlation of expression levels of rhythmic clock and muscle-related genes in the slow muscle

*Note*: The following values were set to define the degree of correlation: data are considered to be moderately correlated if 0.5 < *r* < 0.79 or −0.79 < *r* < −0.5 and there is a strong correlation when *r* ≥ 0.80. And moderate correlation is marked in bold and strong correlation in red color

## Discussion

In the present study, we identified for the first time 15 clock genes, including four transcriptional activation factors (*arntl1*, *arntl2*, *clock* and *npas2*), eight transcriptional repressors (*cry1*, *cry2*, *cry3*, *cry-dash*, *per1*, *per2*, *per3* and *tim*), and three orphan nuclear receptors (*nr1d1*, *nr1d2* and *rorα*) in Chinese perch skeletal muscles. As expected, some of the clock genes exhibited a robust oscillation during the light–dark cycle in the slow or fast skeletal muscle.

The full length cDNA sequences of several key clock genes, such as *clock*, *cry1*, *per1* and *nr1d2*, were cloned from Chinese perch. Silico structural analysis of the deduced amino acid sequences indicated that *clock* and *per1* proteins contain the conserved PAS and bHLH domains [24]. These two domains are required for the circadian clock functions and are highly conserved in different species during evolution [25]. The transcriptional repressor *cry1* has a typical DNA-photolyase and flavin adenine dinucleotide(FAD)-binding domains that are present in all cryptochrome genes [26, 27]. Together with photolyase DNA repair enzymes, FAD-binding domain containing proteins form the cryptochrome/photolyase complex. This active complex has been used in blue light-induced gene expression to affect biological rhythm [26, 28, 29]. In nr1d2 protein*,* two core nuclear domains were also identified from its deduced amino acid sequence. The *nr1d2* binds to *arntl1* and *clock* via a unique zinc finger structure domain to form a complex, which binds and blocks the protein complex formation by Circadian Locomotor Cycles [30, 31].

The daily rhythmicity was observed in many clock genes in fast muscles of several species, indicating a potential regulatory function in muscle physiology and metabolism ([2, 20, 32], and [33]). However, the circadian clock gene expression in slow muscles has not been reported. In the study, we analyzed the *arntl1* and *arntl2* gene expression in Chinese perch and showed that they exhibited a light-biased expression in fast muscles. Several earlier reports in rainbow trout and mouse suggested that the photosensitivity profile in fast muscles is regulated by their homologous expression in either central or peripheral clocks ([10, 34, 35]). However, their dark-biased expression in Chinese perch slow muscle is similar to that observed in Atlantic cod and zebrafish fast muscles. The correlation between *arntl1*/*2* and *clock*/*npas2* in Chinese perch slow muscles indicated that the mechanism underlying the transcriptional activation of the clock system may be similar to those reported from other fish species in slow muscles. *Npas2* which shares a high sequence homology with *clock* protein is able to substitute for *clock* function in the master brain clock and regulates the circadian rhythmicity in the brain. The antiphase profile between *arntl* and *clock* in Chinese perch fast muscles suggested that *arntl* may correlate with other bHLH-PAS factors in fish muscles.

Among the 3 period genes expressed in Chinese perch slow muscles, their dark-biased expression was in agreement with that reported from other fish species, such as goldfish, European sea bass and zebrafish [20, 28, 36, 37]. On the other hand, their light-biased expression in Chinese perch fast muscle is also consistent with the central and peripheral clocks in *Senegalese sole* [24]. The three period genes with the daily rhythmic expression also displayed a positive correlation with the *cry1* gene, the only cryptochrome gene with the daily rhythmicity in Chinese perch fast muscle. Of the period and cry genes with the daily rhythmic expression in Chinese perch slow muscles, *per1/2* showed the positive correlation with both *cry1* and *cry3*. These results suggested that *per* may interact with *cry* to control the transcriptional activation and function in the circadian feedback loop.

*Nr1d1*, *nr1d2* and *rorα* are members of nuclear receptor family, which are involved in stabilizing the circadian clock loop [20, 38–40]. *Nr1d1* and *nr1d2* were identified as the constitutive transcriptional repressors of *arntl1*, whereas *rorα* is the *arntl1* transcriptional activator [2, 41]. In addition, *nr1d1* was considered to be interwoven into the core clock mechanism via downregulating the *clock* expression. As reported in mammals, *Npas2* expression was repressed by these nuclear receptors [40, 42]. In this study, the three nuclear receptors displayed a daily rhythmic expression in Chinese perch fast muscles and *nr1d2* exhibited a negative correlation with the expression of *rorα* gene. This is reflected in their tight regulation with the circadian mechanism. *Nr1d2* also showed a negative correlation with *clock* and *npas2*. Therefore, it is possible that *nrld2* may function by repressing *npas2* and *clock* expression in fast muscles. However, *nr1d2* had no daily rhythmic expression in Chinese perch slow muscles and *nr1d1* showed a direct relationship with the *rorα* expression. It could suggest that Chinese perch slow muscles may have a different circadian mechanism in maintaining the stabilization loop.

In this study, the transcription levels of eleven genes related to myogenesis during the daily cycle were investigated. The data revealed that seven genes had a daily rhythmic expression in fast muscles and five in slow muscles. Myogenic regulatory factors(MRFs) (such as *myoD*, *myf5*, *mrf4* and *myoG*), belong to the same class of helix-loop-helix transcription factors that play distinct and overlapping roles in regulating muscle development and growth [43, 44]. It has been reported that the circadian regulation of *myoD* expression by *lock/arntl* was crucial for the skeletal muscle phenotype and function in mouse [32]. Our study confirmed that *myoD* in Chinese perch fast muscles exhibited a typical daily rhythmicity. Based on our observation, it is possible that the circadian regulation of Chinese perch *myoD* may function in a similar way as reported in mouse. In contrast, we have not obtained any direct evidence for circadian expression of *myoD* in Chinese perch slow muscles. Therefore, it is possible that the differentially expressed MRFs may result from the lineage-specific differences by clock genes. However, our work demonstrated that one of the MRFs, *mrf4,* in Chinese perch slow muscles exhibited a rhythmic expression pattern similar to that described for *myoD* in fast muscles, suggesting that mrf4 may have a potential function in the maintenance of muscle phenotype and function.

## Conclusion

**Fig. 7:**
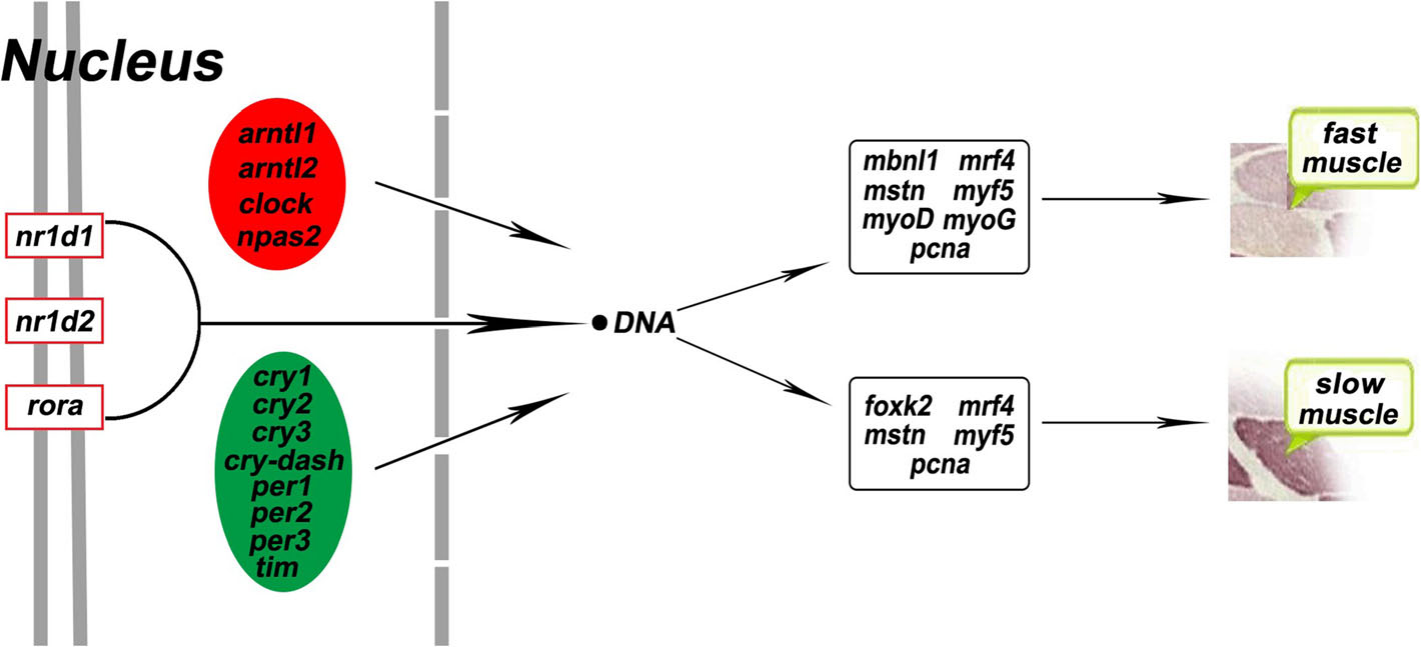
Molecular components of the clock system identified in fast and slow skeletal muscles of Chinese Perch and myogenic genes with daily rhythmic expression. The green and red oval represent genes involved in the peripheral clock components in Chinese Perch skeletal muscles, which is related with the muscle fiber. They comprise members of the transcriptional activator arm (in red: *arntl1*, *arntl2*, *clock* and *npas2*), transcriptional repressor arm (in green: *cry1*, *cry2*, *cry3*, *cry-dash*, *per1*, *per2*, *per3* and *tim*) and nuclei (in nucleus: *nr1d1*, *nr1d2* and *rorα*). The daily rhythmicity of *mbnl1*, *mrf4*, *mstn*, *myf5*, *myoD*, *myoG* and *pcna* play a crucial role in fast muscle specification, and *foxk2*, *mrf4*, *mstn*, *myf5* and *pcna* play an important role in terminal slow muscle differentiation

In this study, we assayed the possible correlation of a functional clock system in Chinese perch slow and fast skeletal muscles. We demonstrated that 10 clock genes and 7 genes related to myogenesis exhibited the daily rhythmicity in fast muscles of Chinese perch. The 11 clock genes and 5 genes related to myogenesis have the daily rhythmicity in slow muscles (Fig. 7). The circadian expression of *mrf4*, *myf5*, *mstn*, and *pcna* may either positively or negatively regulate the transcription of the clock genes in both types of muscles. It is plausible that muscle type-specific maintenance and function is regulated by the core clock genes. This is based on the evidence of daily rhythmicity and apparent correlation of gene expression of clock genes and genes related to myogenesis. Taken together, our data provide new information on the rhythmic expression of clock genes and a better understanding of the circadian clocks in fish muscle phenotype maintaining and function.

## Methods

### Daily rhythm experiment

Adult Chinese perch (body weight 450 ± 10 g) were stocked in 250 m^3^ tanks. Fifteen testing fishes were kept in each tank and a total of six tanks were used in the experiment. To liminate the fish disturbance during sampling, the fish were fed at the same time each day. During the experiment, water temperature was kept at 25 ± 0.8 °C, dissolved oxygen at 85 ± 2% and the light intensity of the water surface at 0.84 W*m^−2^ (200 lx). The testing fishes were acclimated to the above described conditions for 2 weeks during a daily light–dark cycle before sample collection. Six individuals were randomly collected from the each tank in every three hours until 24 h (Zeitgeber time: ZT0, 3, 6, 9, 12, 15, 18, 21 and 24). Sampling of the light treatment at the different time points was basically followed as described by Lazada [2]. Briefly, samples at ZT0 were collected when the light reached to a maximum intensity, the samples at ZT24 were collected when it transited to the light phase; The ZT12 samples were collected at approximately 20 min later, while samples of ZT0-9 and ZT15-24 were collected at the time between the light and dark. The fast muscle was sampled from the dorsal muscle tissue, and slow muscle was collected under the body skin [16]. All of the collected muscle samples were washed with cold and sterilized 1 × PBS to remove contaminating blood and then immediately stored in liquid nitrogen at −80 °C for total RNA extraction.

### Molecular cloning of Chinese perch clock genes

To amplify the cDNA fragments of the clock genes, the primers were designed based on the assembled EST sequences of the Chinese perch muscle database (accession nos: SRX1738860) or the clock gene sequences from several-related fish species (Table 1). The full length cDNAs of the four clock genes were amplified with the SMART RACE cDNA Amplification Kit according to the manufacture’s instruction (Clontech, Palo Alto, CA, USA). Specific nested PCR primers were designed based on the cloned partial sequences (Table 1). For 5′ RACE amplification, the protocols were conducted as follows: 35 cycles of 94 °C for 30 s, 60 °C for 30 s and 72 °C for 1 min. For 3′-RACE, two amplifications were conducted at the same parameters as 5′ RACE the amplification described above.

### Bioinformatic analysis of the clock genes

The cDNA fragment identity was confirmed with BLAST (http://blast.ncbi.nlm.nih.gov) and the deduced amino acid sequences were obtained using the ExPASy Proteomics Server (http://www.expasy.ch). The complete amino acid sequences of the *clock*, *cry1*, *per1* and *nr1d2* domains were analyzed through UniProt (http://www.uniprot.org) and the ExPASy proteomics Server (http://www.expasy.ch). The clock gene sequences of other teleosts were obtained from the NCBI databases and the protein accession numbers are listed in Additional file 2.

### Gene expression analysis

Total RNAs were isolated from Chinese perch muscles using the TRIzol^R^ Reagent (Invitrogen, USA). The RNA samples were treated with RNAse-free DNAse I (Promega, USA) in the presence of RNAse inhibitor (Sigma, China Branch), and then precipitated with ethanol. The obtained RNA was reversely transcribed with Super-Script III RNase H-Reverse Transcriptase (Invitrogen, USA) following the manufacturer’s instruction. For a negative control, no cDNA sample was added in the PCR reaction.

Primers for the qRT-PCR assays were designed using the software Primer 5.0 (Table 7). The reverse transcribed cDNAs from skeletal muscles were used as templates for qRT-PCR assays with SYBR Green PCR reaction kit (Stratagene, Shanghai, China). The qRT-PCR amplification reaction was carried out using the Stratagene Mx3005 system (Stratagene, CA, USA). A total volume of 25 μL reaction was used for the qRT-PCR assays, containing 1 μL cDNA templates, 12.5 μL SYBR Green mix, and 1 μmol/L each of forward and reverse primers. The reaction protocol was used as the standard cycling of the qPCR. Each product identity was verified by dideoxy-mediated chain termination sequencing at Sangon Biotechnology Inc. (Shanghai, China). The relative expression ratio (R) of target mRNA was calculated by R = 2^-ΔΔCt^ [45, 46], where Ct is the cycle threshold. The basic equation employed was ΔΔCt = (Ct _target gene_ − Ct _housekeeping gene_) _experiment_ − (Ct _target gene_ − Ct _housekeeping gene_) _control_. The treanscriptional levels of selected muscle-related genes in Chinese perch fast and slow muscles during a daily cycle were also quantitatively assayed using the same qRT-PCR protocol.

**Table 7:** Primers used for genes cloning and quantitative real-time PCR

Primer name	Sequence (5′-3′)	Items
3’-RACE outer	R:CTGATCTAGAGGTACCGGATCC	3’-RACE
Clock 3’-RACE inner	F:AGACGGCTGTAGTGGCTC	3’-RACE
Clock 3’-RACE inner	F:AGACGGCTGTAGTGGCTC	3’-RACE
5’-RACE outer	F:GACTCGAGTCGACATCG	5’-RACE
Cry1 5’-RACE inner	R: TTGGCATTCATTCTGGGACG	5’-RACE
Cry1 5’-RACE inner	R: GGTCTGGAACCGTTTGTAGG	5’-RACE
Cry1 3’-RACE inner	F: TCAAGGAGACTGGCAAAGCG	3’-RACE
Cry1 3’-RACE inner	F: CCACAAGCCAGCATCAGCAC	3’-RACE
Per1 5’-RACE inner	R: ATTTAGAGTGCTGGCGTGGC	5’-RACE
Per1 5’-RACE inner	R: GGGCGAACCTTCAGATCCTG	5’-RACE
Per1 3’-RACE inner	F: GAGAACGGTGAAACAAATGA	3’-RACE
Per1 3’-RACE inner	F: ACGCTTCACCGAGGAACAGA	3’-RACE
Nr1d2 5’-RACE inner	R: CAGTCTGAAGGGGCAGTGGT	5’-RACE
Nr1d2 5’-RACE inner	R: GACTGGTAGCTGCCGTTGGA	5’-RACE
Nr1d2 3’-RACE inner	F: AGTGCCGCTTCAAGAAATGC	3’-RACE
Nr1d2 3’-RACE inner	F: GGAGATGAGCCTCTTCACTGC	3’-RACE
Arntl1	F: GGCTATCCCTACTCCAACCAG	RT-PCR
Arntl2	R: TTGCTGGGGCTGCTGGAA	RT-PCR
	F: AGGGACCCAAATCGCAAATG	
Clock	R: TGTGGGGAAACAAGGGGAC	RT-PCR
	F: TGCTGGAGGCTCTGGATGG	
Npas2	R: GGTTCTGGTCCACTAAGTCCGTC	RT-PCR
	F: CAGATAGCGAGTTCAGCCAAGA	
Cry1	R: TGGAGAATGAAGGAGCGATGA	RT-PCR
	F: GAATGCCAACTCACTGCTCG	
Cry2	R: CGAAGCAGGGGTTGTTGG	RT-PCR
	F: GAGAAAAGCGTGGGTGGC	
Cry3	R: CTTGCGGTAGAGGTCTGTGAG	RT-PCR
	F: ATCTTGAAGGACTACCCGAACC	
Cry-dash	R: GCTGCCCTCTGCGTGGTTA	RT-PCR
	F: GCCCTGGACCCTCAGCACT	
Per1	R: CCTCTATCCCGATGTTGTTTGG	RT-PCR
	F: CAACAAACTCATCCTCCTGGC	
Per2	R: CGGTGGGTAAACAGGGTAGATT	RT-PCR
	F: TGGTAACGAGTCGCAAGGC	
Per3	R: TCACCAGACTGAAGGCGTTAGA	RT-PCR
	F: CAAAGCCGAGTGAAGGACAG	
Nr1d1	R: GGGTTATCGCTCTGGTTGG	RT-PCR
	F: GCCGTGGTGCTGGTGTCTG	
Nr1d2	R: TTGTTGAGCGTTCGCAGGTC	RT-PCR
	F:TCTCCCCATGTGGACCCTC	
Rorα	R: GGTGCGGTCCTTCACATCG	RT-PCR
	F: GGTGGGTTCTACCTGGACTTCC	
Tim	R: TGAAGGAGCAGTACGGGAAGAA	RT-PCR
	F: GAAGGCTACAGCAAAGACGGA	
Foxk2	R:CTGGCACTTCAGAATGACGGT	RT-PCR
	F: CCTGAGGTGTCTCGGCAAAA	
Mbnl1	R: TGAGCGATGTTGTCTGGAATG	RT-PCR
	F: AGGTGGACAACGGACGGG	
Mrf4	R: CTTTAGGTGGGGAGGAGGGT	RT-PCR
	F: CCGACCTCTGCTGACCATTC	
Mstn	R: GACGCAGAAGACTCACTGGTTT	RT-PCR
	F:GCACATACGCATCCGCTCCCT	
Murf1	R:GTCACGGCCAAGTCATTTCCA	RT-PCR
	F: TCCAGGAACCCCTACCACTACT	
Myf5	R: CACTTCGGCTCTTTGGTGTCTT	RT-PCR
	F:AGGTCAACCACGCTTTCGAG	
myoD	R:GTTTTCCACCTGCTCCCGTA	RT-PCR
	F:CAACGACGCCTTTGAGACCCTG	
myoG	R:GTCCGAATCCCCGCTGTAGTGT	RT-PCR
	F: GGTGTTGGAGTCGGGGTGA	
Pdk4	R: TGGTAACCGTCTTCCTTTTGC	RT-PCR
	F: CTCTGGTGAACATCCGTAATCG	
Pcna	R:ATGGGCTGGGTTCACGCT	RT-PCR
	F: GGACGAGGCGGTCACTATTG	
Ucp3	R: CTGAGGGTGACGGTCTTGGA	RT-PCR
	F: GTATCGGGGAGCGTTTGG	
Rpl-13	R: AGTCCTGCCACCAGTCCGT	internal reference
	F:CACAAGAAGGAGAAGGCTCGGGT	
*β*-actin	R: TTTGGCTCTCTTGGCACGGAT	internal reference
	F:TGCGTGACATCAAGGAGAAGC	
	R:GAGGAAGGAAGGCTGGAAGAG	
hprt1	F:CATACCAAAGCATTACGCAGAAG	internal reference
	R:CACCTCGAATCCTACAAAGTCCG	
rps29	F:TCACCCCAGAAAATTCGGACAGG	internal reference
	R:GTATTTACGGATCAGACCGTGTC	
GAPDH	F:ATCAAGGAAGCGGTGAAGAAGG	internal reference
	R:CGAAGATGGAGGAGTGGGTGTC	
18 S rRNA	F:GGAATGAGCGTATCCTAAACCC	internal reference
	R:CTCCCGAGATCCAACTACAAGC	

The stability of transcription of reference genes was assayed with GeNorm system and total six reference genes were analyzed including Glyceraldehyde-3-phosphate dehydrogenase (GAPDH), *β*-actin, 18 S rRNA gene, hypoxanthine phosphoribosyltransferase 1-like (*hprt1*), epinephelus coioides ribosomal protein S29 (*rps29*) and ribosomal protein L13 (*rpl13*) [47–49]. GeNorm analysis revealed that *rpl13* was the most stable control gene in skeletal muscles (geNorm stability value M = 0.28). The geometric average of these genes were measured by absolute quantification within all samples then calculated by one-way ANOVA procedures and gene expression values are displayed as arbitrary units.

### Statistical analysis

The transcriptional expression levels of the clock and muscle-related genes at each time points were analyzed with the Sigma plot and then were calculated by one-way ANOVA procedures using SPSS software. To compare the difference between the control and experimental groups, Duncan’s multiple range tests were used for the analysis. The differences were considered to be statistically significant when the *P* value was less than 0.05. Data are shown as means ± SE (*n* = 6).

The daily rhythmicity in relation to the expression of the clock and muscle-related genes was assayed with Matlab 7.0 followed as described by earlier studies [2, 37]. To perform a COSINOR analysis, the formula ƒ(t) = M + Acos(t/pi/12 –φ) was used, where ƒ(t) stands for the gene expression level at a given time, mesor (M) stands for the mean value, A stands for is the oscillation amplitude, t is the time in hours and φ stands for the acrophase. The *P* value was defined by the noise/signal of the amplitude (SE(A)/A) and if *P* Value <0.3, the expression levels could considered to display daily rhythmicity [2, 37]. Correlation between the clock and muscle-related gene expression was assayed with Pearson’s correlation test (*r*).

## Additional files

Additional file 1: The 15 clock genes sequences of Chinese perch Genbank accession number. (DOC 36 kb)
Additional file 2: The clock genes protein sequences of other teleosts Genbank accession number. (DOC 39 kb)

## Abbreviations

bHLH: Basic-helix-loop-helix
FAD: Flavin adenine dinucleotide
GAPDH: Glyceraldehyde-3-phosphate dehydrogenase
*L. crocea*: *Larimichthys crocea*
MFMR: Multifunctional mosaic region
MRFs: Myogenic regulatory factors
MS-222: Tricaine methanesulfonate
ORF: Open reading frame
PAS: Per-Arnt-Ser
UTR: Untranslated regions
ZT: Zeitgeber time

## References

[1] Wenger CB. The regulation of body temperature. In: Rhoades RA, Tanner GA, editors. Medical Physiology. New York: Little, Brown; 1995: 587-613

[2] Lazado CC, Kumaratunga HP, Nagasawa K, Babiak I, Giannetto A, Fernandes JM (2014). Daily rhythmicity of clock gene transcripts in Atlantic cod fast skeletal muscle. PLoS One.

[3] Kojima S, Shingle DL, Green CB (2011). Post-transcriptional control of circadian rhythms. J Cell Sci.

[4] Lowrey PL, Takahashi JS (2004). Mammalian circadian biology: elucidating genome-wide levels of temporal organization. Annu Rev Genomics Hum Genet.

[5] Ueda HR, Hayashi S, Chen W, Sano M, Machida M, Shigeyoshi Y, Iino M, Hashimoto S (2005). System-level identification of transcriptional circuits underlying mammalian circadian clocks. Nat Genet.

[6] Ptitsyn AA, Zvonic S, Conrad SA, Scott LK, Mynatt RL, Gimble JM (2006). Circadian clocks are resounding in peripheral tissues. PLoS Comput Biol.

[7] Hogenesch JB, Gu YZ, Jain S, Bradfield CA (1998). The basic-helix–loop–helix-PAS orphan MOP3 forms transcriptionally active complexes with circadian and hypoxia factors. Proc Natl Acad Sci.

[8] Froy O (2010). Metabolism and circadian rhythms—implications for obesity. Endocr Rev.

[9] Braun T, Gautel M (2011). Transcriptional mechanisms regulating skeletal muscle differentiation, growth and homeostasis. Nat Rev Mol Cell Biol.

[10] McCarthy JJ, Andrews JL, McDearmon EL, Campbell KS, Barber BK, Miller BH, Walker JR, Hogenesch JB, Takahashi JS, Esser KA (2007). Identification of the circadian transcriptome in adult mouse skeletal muscle. Physiol Genomics.

[11] Ahammad AS, Asaduzzaman M, Asakawa S, Watabe S, Kinoshita S (2015). Regulation of gene expression mediating indeterminate muscle growth in teleosts. Mech Dev.

[12] Thompson A, Vo D, Comfort C, Zakon HH (2014). Expression evolution facilitated the convergent neofunctionalization of a sodium channel gene. Mol Biol Evol.

[13] Johnston I, Camm J-P (1987). Muscle structure and differentiation in pelagic and demersal stages of the Antarctic teleost Notothenia neglecta. Mar Biol.

[14] Van Raamsdonk W, Pool C, de Kronnie G (1978). Differentiation of muscle fiber types in the teleost Brachydanio rerio. Anat Embryol.

[15] Barton-Davis ER, Shoturma DI, Musaro A, Rosenthal N, Sweeney HL (1998). Viral mediated expression of insulin-like growth factor I blocks the aging-related loss of skeletal muscle function. Proc Natl Acad Sci.

[16] Bone Q (1966). On the function of the two types of myotomal muscle fibre in elasmobranch fish. J Mar Biol Ass UK.

[17] Lin J, Wu H, Tarr PT, Zhang C-Y, Wu Z, Boss O, Michael LF, Puigserver P, Isotani E, Olson EN (2002). Transcriptional co-activator PGC-1α drives the formation of slow-twitch muscle fibres. Nature.

[18] De Almeida-Val VMF, Gomes AC, Lopes NP (2005). Metabolic and physiological adjustments to low oxygen and high temperature in fishes of the Amazon. Fish Physiol.

[19] Whitmore D, Foulkes NS, Strähle U, Sassone-Corsi P (1998). Zebrafish Clock rhythmic expression reveals independent peripheral circadian oscillators. Nat Neurosci.

[20] Amaral IP, Johnston IA (2012). Circadian expression of clock and putative clock-controlled genes in skeletal muscle of the zebrafish. Am J Physiol Regul Integr Comp Physiol.

[21] Amaral IP, Johnston IA (2012). Experimental selection for body size at age modifies early life-history traits and muscle gene expression in adult zebrafish. J Exp Biol.

[22] Chu WY, Liu LS, Li YL, Chen L, Wang KZ, Li HH, Du SJ, Zhang JS (2013). Systematic identification and differential expression profiling of microRNAs from white and red muscles of *Siniperca chuatsi*. Curr Mol Med.

[23] Tang D, Andrews M, Cobcroft JM (2007). The first chondracanthid (Copepoda: Cyclopoida) reported from cultured finfish, with a revised key to the species of *Chondracanthus*. J Parasitol.

[24] Martín-Robles ÁJ, Whitmore D, Sánchez-Vázquez FJ, Pendón C, Muñoz-Cueto JA (2012). Cloning, tissue expression pattern and daily rhythms of Period1, Period2, and Clock transcripts in the flatfish Senegalese sole, *Solea senegalensis*. J Comp Physiol B.

[25] Hirayama J, Sassone-Corsi P (2005). Structural and functional features of transcription factors controlling the circadian clock. Curr Opin Genet Dev.

[26] Öztürk N, Song S-H, Özgür S, Selby C, Morrison L, Partch C, Zhong D, Sancar A. Structure and function of animal cryptochromes. In: Cold Spring Harbor Symposia on Quantitative Biology: 2007. New York; Cold Spring Harbor Laboratory Press. 2007. p. 119–31. 10.1101/sqb.2007.72.015 18419269

[27] Busza A, Emery-Le M, Rosbash M, Emery P (2004). Roles of the two *Drosophila* CRYPTOCHROME structural domains in circadian photoreception. Science.

[28] del Pozo A, Vera LM, Sánchez JA, Sánchez-Vázquez FJ (2012). Molecular cloning, tissue distribution and daily expression of cry1 and cry2 clock genes in European seabass (*Dicentrarchus labrax*). Comp Biochem Physiol A Mol Integr Physiol.

[29] Brudler R, Hitomi K, Daiyasu H, Toh H, Kucho K-i, Ishiura M, Kanehisa M, Roberts VA, Todo T, Tainer JA (2003). Identification of a new cryptochrome class: structure, function, and evolution. Mol Cell.

[30] Zhang EE, Liu AC, Hirota T, Miraglia LJ, Welch G, Pongsawakul PY, Liu X, Atwood A, Huss JW, Janes J (2009). A genome-wide RNAi screen for modifiers of the circadian clock in human cells. Cell.

[31] Cheng H-YM, Papp JW, Varlamova O, Dziema H, Russell B, Curfman JP, Nakazawa T, Shimizu K, Okamura H, Impey S (2007). microRNA modulation of circadian-clock period and entrainment. Neuron.

[32] Andrews JL, Zhang X, McCarthy JJ, McDearmon EL, Hornberger TA, Russell B, Campbell KS, Arbogast S, Reid MB, Walker JR (2010). CLOCK and BMAL1 regulate MyoD and are necessary for maintenance of skeletal muscle phenotype and function. Proc Natl Acad Sci.

[33] Lazado CC, Nagasawa K, Babiak I, Kumaratunga HP, Fernandes JM (2014). Circadian rhythmicity and photic plasticity of myosin gene transcription in fast skeletal muscle of Atlantic cod (*Gadus morhua*). Mar genomics.

[34] Patiño MAL, Rodríguez-Illamola A, Conde-Sieira M, Soengas JL, Míguez JM (2011). Daily rhythmic expression patterns of clock1a, bmal1, and per1 genes in retina and hypothalamus of the rainbow trout, *Oncorhynchus mykiss*. Chronobiol Int.

[35] Davie A, Sanchez JA, Vera LM, Sanchez-Vazquez J, Migaud H (2011). Ontogeny of the circadian system during embryogenesis in rainbow trout (*Oncorhynchus mykyss*) and the effect of prolonged exposure to continuous illumination on daily rhythms of per1, clock, and aanat2 expression. Chronobiol Int.

[36] McStay E, Migaud H, Vera LM, Sánchez-Vázquez FJ, Davie A (2014). Comparative study of pineal clock gene and AANAT2 expression in relation to melatonin synthesis in Atlantic salmon (Salmo salar) and European seabass (*Dicentrarchus labrax*). Comp Biochem Physiol A Mol Integr Physiol.

[37] Velarde E, Haque R, Iuvone P, Azpeleta C, Alonso-Gómez A, Delgado M (2009). Circadian clock genes of goldfish, *Carassius auratus*: cDNA cloning and rhythmic expression of period and cryptochrome transcripts in retina, liver, and gut. J Biol Rhythms.

[38] Vatine G, Vallone D, Gothilf Y, Foulkes NS (2011). It’s time to swim! Zebrafish and the circadian clock. Febs Letters.

[39] Emery P, Reppert SM (2004). A rhythmic Ror. Neuron.

[40] Raghuram S, Stayrook KR, Huang P, Rogers PM, Nosie AK, McClure DB, Burris LL, Khorasanizadeh S, Burris TP, Rastinejad F (2007). Identification of heme as the ligand for the orphan nuclear receptors REV-ERBα and REV-ERBβ. Nat Struct Mol Biol.

[41] Mazzoccoli G, Tomanin R, Mazza T, D’Avanzo F, Salvalaio M, Rigon L, Zanetti A, Pazienza V, Francavilla M, Giuliani F (2013). Circadian transcriptome analysis in human fibroblasts from Hunter syndrome and impact of iduronate-2-sulfatase treatment. BMC Med Genomics.

[42] Baggs JE, Price TS, DiTacchio L, Panda S, FitzGerald GA, Hogenesch JB (2009). Network features of the mammalian circadian clock. PLoS Biol.

[43] Naidu PS, Ludolph DC, To RQ, Hinterberger TJ, Konieczny SF (1995). Myogenin and MEF2 function synergistically to activate the MRF4 promoter during myogenesis. Mol Cell Biol.

[44] Chong S-W, Nguyet L-M, Jiang Y-J, Korzh V (2007). The chemokine Sdf-1 and its receptor Cxcr4 are required for formation of muscle in zebrafish. BMC Dev Biol.

[45] Bustin SA, Benes V, Garson JA, Hellemans J, Huggett J, Kubista M, Mueller R, Nolan T, Pfaffl MW, Shipley GL (2009). The MIQE guidelines: minimum information for publication of quantitative real-time PCR experiments. Clin Chem.

[46] Livak KJ, Schmittgen TD (2001). Analysis of relative gene expression data using real-time quantitative PCR and the 2− ΔΔCT method. Methods.

[47] Zhou RX, Meng T, Meng HB, Cheng DX, Bin SY, Cheng J, Fu GH, Chu WY, Zhang JS (2010). Selection of reference genes in transcription analysis of gene expression of the Mandarin fish, *Siniperca chuasti*. Dongwuxue Yanjiu.

[48] Liu J, Wang Q, Sun M, Zhu L, Yang M, Zhao Y (2014). Selection of reference genes for quantitative real-time PCR normalization in panax ginseng at different stages of growth and in different organs. PLoS One.

[49] Sun JH, Nan LH, Gao CR, Wang YY (2012). Validation of reference genes for estimating wound age in contused rat skeletal muscle by quantitative real-time PCR. Int J Leg Med.

