# Supplementary material for: Daily rhythmicity of clock gene transcript levels in fast and slow muscle fibers from Chinese perch (Siniperca chuatsi)
Source: BMC Genomics. 2016 Dec 8;17:1008. doi: 10.1186/s12864-016-3373-z (PMC5146901; doi:10.1186/s12864-016-3373-z)
Supplement: Additional file 1: — The 15 clock genes sequences of Chinese perch Genbank accession number. (DOC 36 kb) [file 12864_2016_3373_MOESM1_ESM.doc]

**Additional files**

**Additional file 1.** The 15 clock genes sequences of Chinese perch Genbank accession number

| Gene name | Accession number |
| --- | --- |
| arntl1 | KP702269 |
| arntl2 | KP702270 |
| cry1 | KP702272 |
| cry2 | KP702273 |
| cry3 | KP702274 |
| npas2 | KP702276 |
| nr1d1 | KP702278 |
| nr1d2 | KP702279 |
| per1 | KP702280 |
| per2 | KP702281 |
| per3 | KP702282 |
| rorα | KP702283 |
| tim | KP702284 |
| clock | KT895225 |
| cry-dash | KP702275 |
