# Supplementary material for: Daily rhythmicity of clock gene transcript levels in fast and slow muscle fibers from Chinese perch (Siniperca chuatsi)
Source: BMC Genomics. 2016 Dec 8;17:1008. doi: 10.1186/s12864-016-3373-z (PMC5146901; doi:10.1186/s12864-016-3373-z)
Supplement: Additional file 2: — The clock genes protein sequences of other teleosts Genbank accession number. (DOC 39 kb) [file 12864_2016_3373_MOESM2_ESM.doc]

**Additional file 2.** The clock genes protein sequences of other teleosts Genbank accession number

| Species’ gene name | Accession number |
| --- | --- |
| *Danio reria* clcok | AAD27749.1 |
| *Larimichthys crocea* clock | XP_010754400.1 |
| [***Notothenia***](http://www.baidu.com/link?url=48z8Y8L6q1w3COS-8QiP_g-VeMyMVS9pv89G3A5hUXeAc2kt7uuIfHzSL7-BoSdJx2Ox9jqaXeSC4cL4VXQ-iK) *coriiceps* clock | XP_010795869.1 |
| *Danio reria* cry1 | NP_001070765.2 |
| *Larimichthys crocea* cry1 | KKF14195.1 |
| [***Stegastes partitus***](http://www.baidu.com/link?url=nWm6WcVtvEC6qnX1Xlbp0Zu7vW42quCRWKx8udyiZN1s4ZGdewbfqebpX1aJRBM64bqJ4D32a7MEeL7inIhsga)cry1 | XP_008279107.1 |
| *Danio reria* per1 | NP_997604.2 |
| *Larimichthys crocea* per1 | XP_010751195.1 |
| [***Dicentrarchus labrax***](https://www.baidu.com/link?url=X6MIGRJ6x3bcd4T5-3-3T7NhySh9ZlUMe9Dkze_ITRF0DZFsTicTQzD9XHv-cq7TFJkBCfQkTxz-_BvnIFOmdaqtM94LdJmuGl8g21ZIJxC&wd=&eqid=9b32700500019ba30000000557e25423)per1 | ADI71975.1 |
| *Danio reria* nr1d2 | NP_571140.1 |
| *Larimichthys crocea* nr1d2 | KKF32860.1 |
| [*Stegastes partitus*](http://www.baidu.com/link?url=nWm6WcVtvEC6qnX1Xlbp0Zu7vW42quCRWKx8udyiZN1s4ZGdewbfqebpX1aJRBM64bqJ4D32a7MEeL7inIhsga)nr1d2 | XP_008293875.1 |
